# Preliminary Evaluation of Cement Mortars Containing Waste Silt Optimized with the Design of Experiments Method

**DOI:** 10.3390/ma14030528

**Published:** 2021-01-22

**Authors:** Abbas Solouki, Giovanni Viscomi, Piergiorgio Tataranni, Cesare Sangiorgi

**Affiliations:** 1 Department of Civil, Chemical, Environmental and Materials Engineering, University of Bologna, 40136 Bologna, Italy; Cesare.sangiorgi4@unibo.it; 2 S.A.P.A.B.A. srl (Società Anonima Prodotti Asfaltico Bituminosi Affini), 40037 Pontecchio Marconi, Italy; g.viscomi@sapaba.it

**Keywords:** cement mortar, DOE, waste silt, quarry waste, mixture design, aggregate recycling

## Abstract

Every year, up to 3 billion tons of non-renewable natural aggregates are demanded by the construction sector and approximately 623 million tons of waste (mining and quarrying) was produced in 2018. Global efforts have been made to reduce the number of virgin aggregates used for construction and infrastructure sectors. According to the revised waste framework directive in Europe, recycling at least 70% of construction and demolition waste materials by 2020 was obligatory for all member states. Nonetheless, quarries must work at full capacity to keep up with the demands, which has made quarry/mining waste management an important aspect during the past decades. Amongst the various recycling methods, quarry waste can be included in cement mortar mixtures. Thus, the current research focuses on producing cement mortars by partially substituting natural sand with the waste silt obtained from the limestone aggregate production in S.A.P.A.B.A. s.r.l. (Italy). A Design of Experiments (DOE) method is proposed to define the optimum mix design, aiming to include waste silt in cement mortar mixtures without affecting the final performance. Three cement mortar beams were produced and tested for each of the 49 randomized mixtures defined by the DOE method. The obtained results validate the design approach and suggest the possibility of substituting up to 20% of natural sand with waste silt in cement mortar mixtures.

## 1. Introduction

The first step in every quarrying process starts with the stripping stage, where the unwanted materials are removed from the Earth’s surface. Based on the quality and type of the rocks, up to three different crushing stages could be applied during aggregate production. At the final step, the crushed aggregates go through the washing and screening stage, which is vital for quality and gradation control of the materials. The water used during the washing process is directed to precipitation tanks for further processing. The resulting residues which mainly consist of dirt and very fine powdery substances are piped out into artificial lakes and ponds called tailing mines or sedimentation lakes. These materials, mostly mineral fillers from plant processes, could become an environmental issue due to the landfilling limitations and strict legislation on their disposal [1]. Every year, up to 3 billion tons of non-renewable natural aggregates are demanded by the construction sector [2] and approximately 623 million tons of waste (mining and quarrying) were produced in 2018 [3]. Global efforts have been made to reduce the number of virgin aggregates used for construction and infrastructure sectors. For instance, based on the revised waste framework directive in Europe recycling of at least 70% of construction and demolition waste materials by 2020 was obligatory for all member states [2]. Nonetheless, quarries must work at full capacity to keep up with the demand for raw materials. Therefore, quarry/mining waste management has become an important aspect during the past decades and various methods have been proposed for reducing its impacts on the environment.

Quarry waste has been used in various applications such as geopolymer production [4], soil stabilization [5], pavements [6,7] and production of artificial aggregates [8]. Studies and experimental applications have also highlighted the possibility of using quarry waste in cementitious materials. For instance, Cavaleri et al. (2018) partially substituted sand with quarry dust to produce concrete. The results indicated that the substitution of 13% of sand with limestone quarry dust could improve the mechanistic properties of the cement, whereas 26% of quarry dust led to lower mechanical properties [9]. The inclusion of quarry dust in cement bound materials and its effect on various properties of cement such as transport, dimensional stability, alkali–silica reaction, the heat of hydration and color were investigated. Medina et al. [10] found no adverse effects from the addition of quarry dust into the mixture and suggested its use in the design of new type II/A conventional as well as type II/A special low heat cement. These alternative applications could potentially reduce the environmental impact of using raw materials and of course decrease the impact of landfilling industrial waste fillers. Felekoglu (2007) incorporated quarry dust limestone powder in Self-Compacting Concrete (SCC) and paste applications with the aim of reducing the environmental impact of the waste quarry powder [11]. Various physical and mechanical properties of the cement paste were examined, and the performance of the mixture indicated the possibility of using up to 10% of limestone quarry dust in normal-strength self-compacting cement. Similar work on self-compacting concrete was carried on by Uysal et al. [12], where Portland cement was partially replaced with different quarry dust including limestone, basalt, and marble. The results were similar to previous studies which suggested the economic feasibility of using the mentioned powders in SCC production [11,12]. In a different attempt to manage mine waste, a study was conducted aiming to investigate the possibility of using gold waste rocks as construction materials [13]. The waste materials were collected from a gold open-mine site located in the Abitibi-Temiscamingue region (QC, Quebec, Canada) and the material was used for concrete production. The obtained compressive strength for concretes made with waste rocks and natural sand and gravel were comparable after 28 and 56 days of curing [13]. In a separate study, limestone dust was substituted by 30% of the total concrete weight to produce lightweight concrete. The mechanical properties of the synthetic material were tested and the result showed acceptable performance [14]. However, the authors indicated that further work and tests regarding the mechanical properties of the lightweight concrete are required.

Concrete and mortars are produced using different design methods. For instance, various common approaches have been reviewed for the production of self-compacting cement [15]. the mixture could be designed based on empirical methods. Compressive strength or rheological properties of the paste could also determine the mixture design. However, an approach that has not been fully exploited is the use of statistical models for concrete mixture design. For instance, the effect of different ingredients such as glass fiber, metakaolin (MK), paste and silica sand on the flexural and compressive strength of glass fiber (GF)-reinforced concrete (GRC) was studied [16]. The authors also applied the Taguchi method to optimize the final mix design based on four factors including aggregate (SS)-to-cement ratio (A/C), GF, W/C and MK contents. The data indicated that SS, MK, GF and CP contents significantly affected the final strength of the concrete mixtures. However, most statistical approaches are limited to factorial designs [17,18] and response surface methods [19,20] and less attention has been paid to mixture-design of experiments methods. It is by the use of a mixture-design approach that one could simultaneously study the effects of all the components and their interactions. The effect of including fly ash (FA), nano-silica (nS), and recycled plastic on the mechanical properties and cost of concrete was investigated by applying two different mixture design approaches [21]. The first model was produced from a screening model and the results were then used as an input for the sequential optimization. Based on the models, the authors suggested that by adding 2.5% of nS and 10% of FA about 44% of coarse aggregates could be substituted by plastic. A simple lattice mixture design having three factors and five levels was applied to study the effect of three different sand types on self-compacting cement properties [22]. The models indicated an increase in compressive strength with an increase in crushed sand proportions. However, when dune sand was used, the resulting strength values dropped. 

Transforming quarry dust/waste into secondary materials could give a second chance to waste materials, contribute to waste management, balance natural resource utilization and produce a circular economy for the construction sector [23,24,25,26]. Thus, the current research focused on producing cement mortars by substituting natural sand with the waste silt obtained from limestone aggregate production S.A.P.A.B.A. s.r.l. (Pontecchio Marconi, Italy).

## 2. Materials

**Figure 1:**
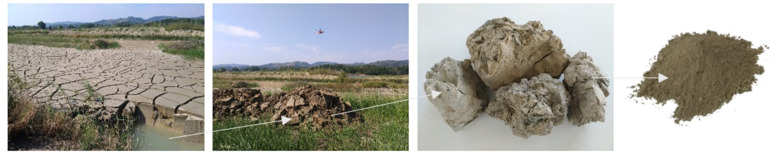
Silt obtained from sedimentation lakes at S.A.P.A.B.A. spa, Bologna (Italy).

Limestone aggregates are generally excavated from loose earth and transported to the production plant in S.A.P.A.B.A s.r.l. (Società Anonima Prodotti Asfaltico Bituminosi Affini) During the washing process, unwanted substances such as silt and clay are separated from the limestone aggregates. The silty materials are then piped and landfilled in the sedimentation lakes. For the current study, the silt was excavated from S.A.P.A.B.A.’s lakes, dried, crushed, sieved and stored in sealed plastic bags for further use (Figure 1).

**Figure 2:**
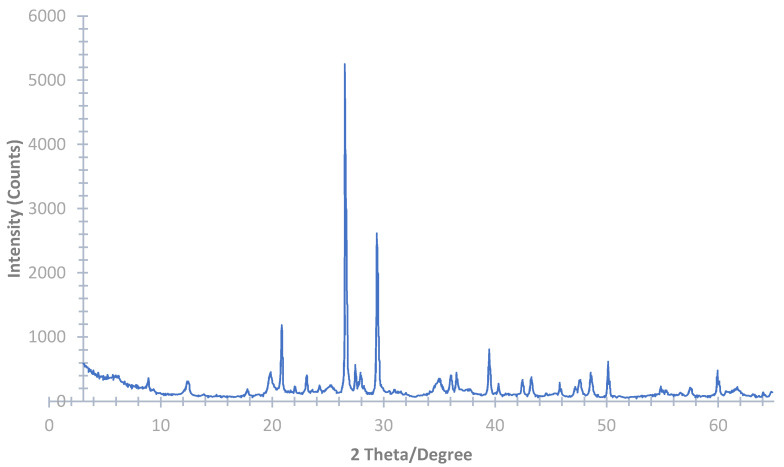
Waste silt XRD analysis revealing a crystalline structure observed as sharp peaks in the diagram.

The elemental and compound analysis of the waste silt was examined using X-ray fluorescence (XRF) (Shimadzu, Kyoto, Japan) and X-ray powder diffraction (XRD) (Rigaku, Tokyo, Japan), respectively. The raw material had a pH of 7.0, where almost 60% of the chemical composition is made up of SiO_2_ and Al_2_O_3_ (Table 1). Moreover, the number of hazardous materials such as arsenic, barium, beryllium, cadmium, cobalt, chrome, mercury, nickel, lead and copper were within the Italian legal limits. The mineralogical evaluation (Figure 2) revealed the presence of quartz (32%), calcite (28%), illite/micas (21%), chlorite (5%) with traces of dolomite and feldspars.

**Figure 3:**
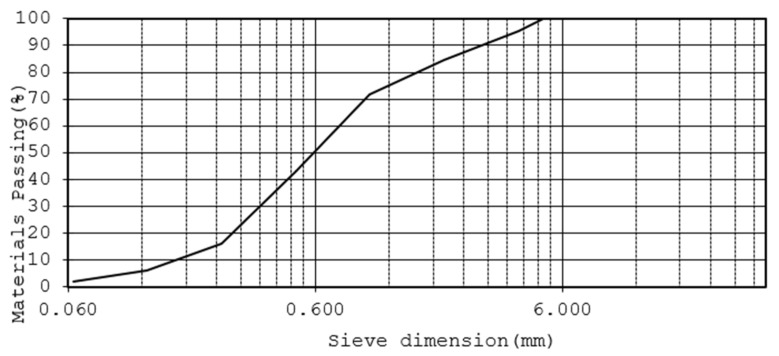
Limestone sand gradation curve.

Natural limestone sand (0–4 mm) was used for the cement mortar production. The gradation curve is shown in Figure 3. The obtained values for bulk density (g/cm^3^) (UNI 1097-6), sand equivalent (uni 933-8) and harmful fines (gMB/KG) (UNI 933-9) were reported as 2.658, 90 and 0.5, respectively. The light organic impurities were reported as 0.07 which was well below the 0.5 limits. Finally, a 42.5 R white Portland cement (Buzzi Unicem, Casale Monferrato, Italy) and a specially designed additive that allows the use of swelling clays or aggregates with very high fines were used.

## 3. Methods

### 3.1. Design of Experiments

In every research or discovery process, the development of experiments is fundamental to achieving results. Each experiment has a determined number of variables with a certain number of levels, where the effect of each variable is investigated on desirable outcomes. Scientists have been using experimental design methods to fit data to empirical functions and provide information about a system or a process. Experimental designs are an approach that facilitates the selection of variables and levels in such a way that the variations and errors are minimized.

As for the first step in any Design of Experiments (DOE), the purpose of the experiment should be determined. In some cases, optimization of a process or response(s) is the main goal, whereas in some studies the effect of certain factors and their levels on the outcome is the focus. Therefore, each experiment and DOE will have a specific purpose and need to be tackled differently. The boundaries of an experiment are defined and restrained based on selected ranges of the variables. For instance, selecting a testing temperature between 20 to 80 °C for a specific experiment will define its experimental region. The design will be finalized by determining the number of levels for the corresponding factors. 

A Response Surface Methodology (RSM) [27] is a statistical tool, which investigates the relationship between variables of an experiment with the produced outcomes. Compared to a full factorial design, RSM can significantly reduce the number of required experimental runs. In many studies, the aim is to select the ingredient’s proportion of a blend. In such cases, the implementation of a mixture design (a special type of RSM) could effectively determine the dependent variables’ proportions that will produce the desired response. In a mixture design experiment, the dependent factors are proportions of different components of a blend and the total sum of all factors for each run will be equal to 1 or 100% (Equation (1)). The mixture DOE allows studying the effects of different ingredients/factors on different responses such as compressive and flexural strength of the mortar specimens. However, in such cases, applying standard design will require a higher number of experiments to find the final mix design. An *n*-component mixture is presented in Equation (1).

(1) $$0\leq x_{i}\leq 1\;i=1,\;2,\;3,\;\ldots ,\;n\;\overset{n}{\underset{i=1}{\sum }}x_{i}=1\;\;$$

where the proportion of the ith ingredient/factor is represented by *x_i_*.

Two of the most widely used mixture designs are the simplex lattice and simplex centroid designs. The former design is applied when the components/ingredients are equally spaced within the design. Furthermore, in such cases, a full cubic analysis could be applied. However, only special cubic estimates could be conducted for centroid design. In both cases, no constraints or boundary limits are set. On the other hand, an extreme vertex design could only be used when linear or boundary constraints are set for the components. In such cases, the mixture design will only cover a small proportion within the simplex design and usually occurs when components have upper and lower boundaries. 

Each DOE has its specific requirements and inputs. For the current study, a mixture DOE having 5 different components including cement, water, sand, silt and additive was proposed. Due to the high number of factors, the extreme vertices design was used rather than the simplex centroid design. Extreme vertices design only covers small spaces in the simplex design and could be adapted when the design space is not linear. However, not much literature has been found regarding extreme vertices design for cement bound materials [16,21]. 

To produce the mixture DOE, the five components and their corresponding boundaries were inputted into JMP^®^ software. The determined upper and lower boundaries (total weight of mixture) for cement, water, sand, silt and additive were as 22–28%, 12–20%, 43–61%, 2–20% and 0.17–0.35%, respectively. As mentioned above extreme vertices design was selected. Consequently, a DOE having 49 randomized runs was produced, where for each run 3 specimens from the same batch were produced. The average value for the corresponding strength (compressive or flexural) was used as inputs to produce the models. Therefore, after inputting the flexural strength (FS) and Unconfined Compressive Strength (UCS) values in the DOE, two corresponding models were produced. The data analysis was also carried out in JMP^®^ software (Version 14.0. SAS Institute Inc., Cary, NC, USA, 1989–2019). Up to five levels of interactions were chosen and the forward selection with Akaike Information Criteria (AIC) was applied to determine the significance of the models. The model was produced, and the optimum results were indicated using the prediction profiler tool for both compressive and flexural strength measurements. The desirability factor was based on the maximum usage of silt in the mixture. Therefore, the value of the silt was set to 20% and the corresponding values for the remaining components were calculated according to the obtained models.

### 3.2. Sample Preparation

**Figure 4:**
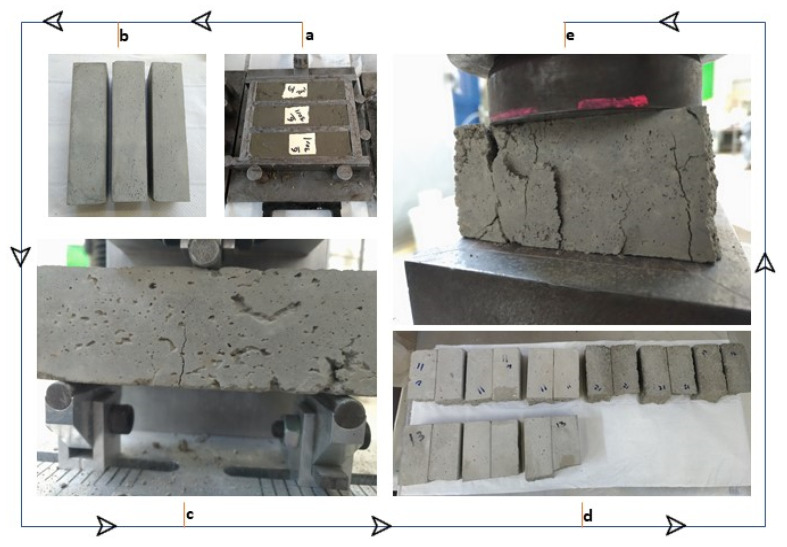
Cement mortar sample production and strength (flexural and compressive) testing procedure.

The samples were prepared by mixing Cement, sand and silt before adding water and the additive (Figure 4). The mortar was poured into metal molds (4 × 4 × 16 cm) and was cured at room temperature for 28 days. The samples were first tested for flexural strength resulting in each beam to be broken into half. Each half was then tested for compressive strength. The whole procedure was based on EN 1015-11:2019 (E) standard. 

## 4. Results and Discussion

An efficient way of studying a mixture in which all components sum to 1 (100%) is to use the mixture design approach. With this method, the changes in the ingredients of a mixture or blend and the resulting effects on the responses could be explored. Thus, by applying the mixture design concept, the effect of cement, water, sand, silt and the additive on the responses including compressive and flexural strength were investigated. 

**Figure 5:**
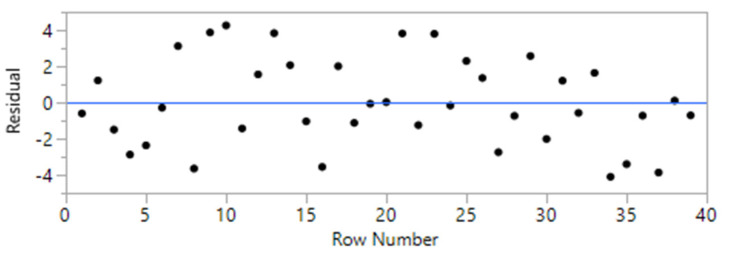
Residual plot for UCS model.

**Figure 6:**
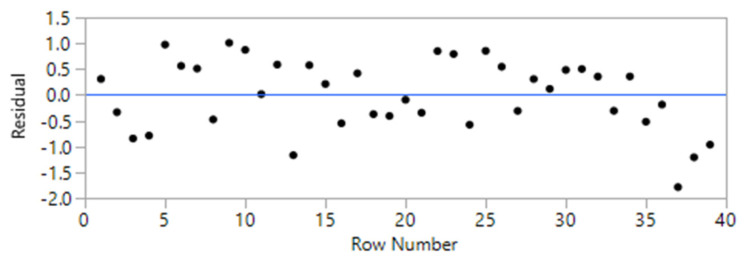
Residual plot for the flexural strength model.

The initial model was constructed based on 49 randomized runs. The order of the tests was randomized to reduce the chance of bias in the results that could have occurred due to differences in materials or experimental conditions. However, to improve the accuracy of the models and based on the residual plots, a total of 10 runs were identified as outliers and were eliminated from the model [28]. Consequently, the second attempt improved the overall accuracy of the models up to 20%. The summary of fit and the residual plots for the models are presented in Table 2 and Table 3 and Figure 5 and Figure 6. The results indicated an R^2^ value of 95.5 and 91.2% for the models related to UCS and FS, respectively. 

**Figure 7:**
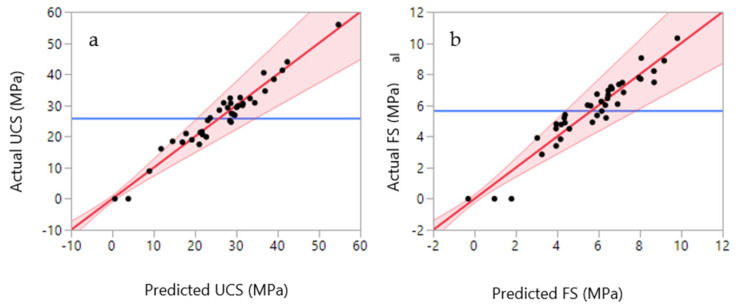
Actual versus predicted values for: (**a**) compressive; (**b**) flexural strength.

The design of experiments consisting of 39 randomized runs is presented in Table 4. The flexural and UCS were inputted into the DOE resulting in a model (equation). For each of the 39 runs, the obtained model was used to calculate the FS and UCS, which are also included in the table as predicted FS and predicted UCS. The actual versus the predicted values for flexural and UCS are also depicted in Figure 7, where the blue line indicates the average value for both UCS and FS. The graphs indicate a linear relationship between actual and predicted values which is also backed up by the high reliability of the models (R^2^). The calculated models for UCS and FS are presented as Supplementary Data.

### 4.1. Compressive Strength

The analysis of variance for the UCS predicted model is shown in Table 5. Based on the analysis, a change in the amounts of components (cement, water, sand, silt, additives) significantly (*p* < 0.0001) affects the compressive strength of the cement mortars. The t-ratio reported in Table 6 indicates the influence of each component on the resulting compressive strength values. Compared to other main components, the sand has the highest effect on UCS followed by cement and silt. Thus, the maximized value for the responses will be obtained by maximizing the sand component in the mixture. On the other hand, an increase in water amount could decrease the UCS value which is shown as a negative ratio of −1.63 in Table 6. The water and additive did not show a significant effect on the compressive strength (*p* ≰ 0.05). However, despite their insignificance, the water and additive were not deleted from the model. It must be noted that components/ingredients could not be deleted in a mixture design process since the resulting mixture will be different than the initial one. For instance, if cement is removed from the mix, the resulting product will no longer be a cement mortar. It is also noteworthy to mention that the main components in Table 6 and Table 8 were coded as pseudo-components. This approach simplifies design construction and model fitting and makes parameter estimates more meaningful.

**Figure 8:**
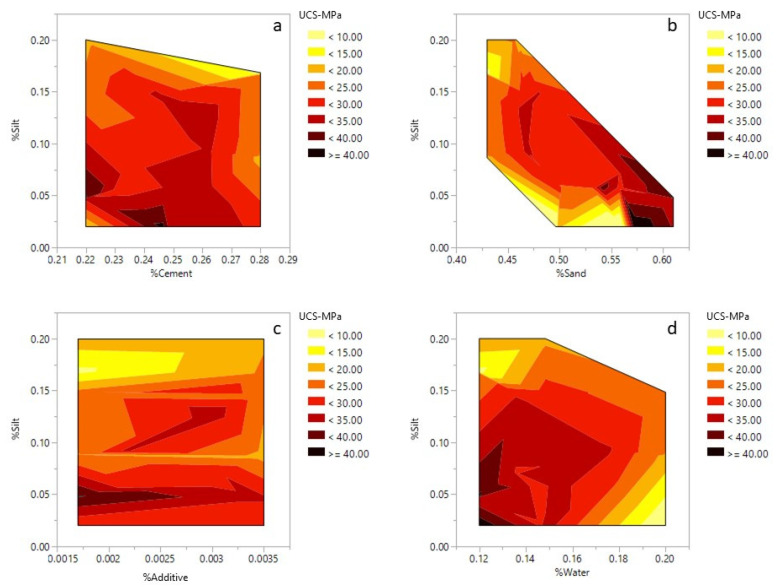
Contour plots for 28 days compressive strength: (**a**) silt–cement; (**b**) silt–sand; (**c**) silt–admixture; (**d**) silt–water.

**Figure 9:**
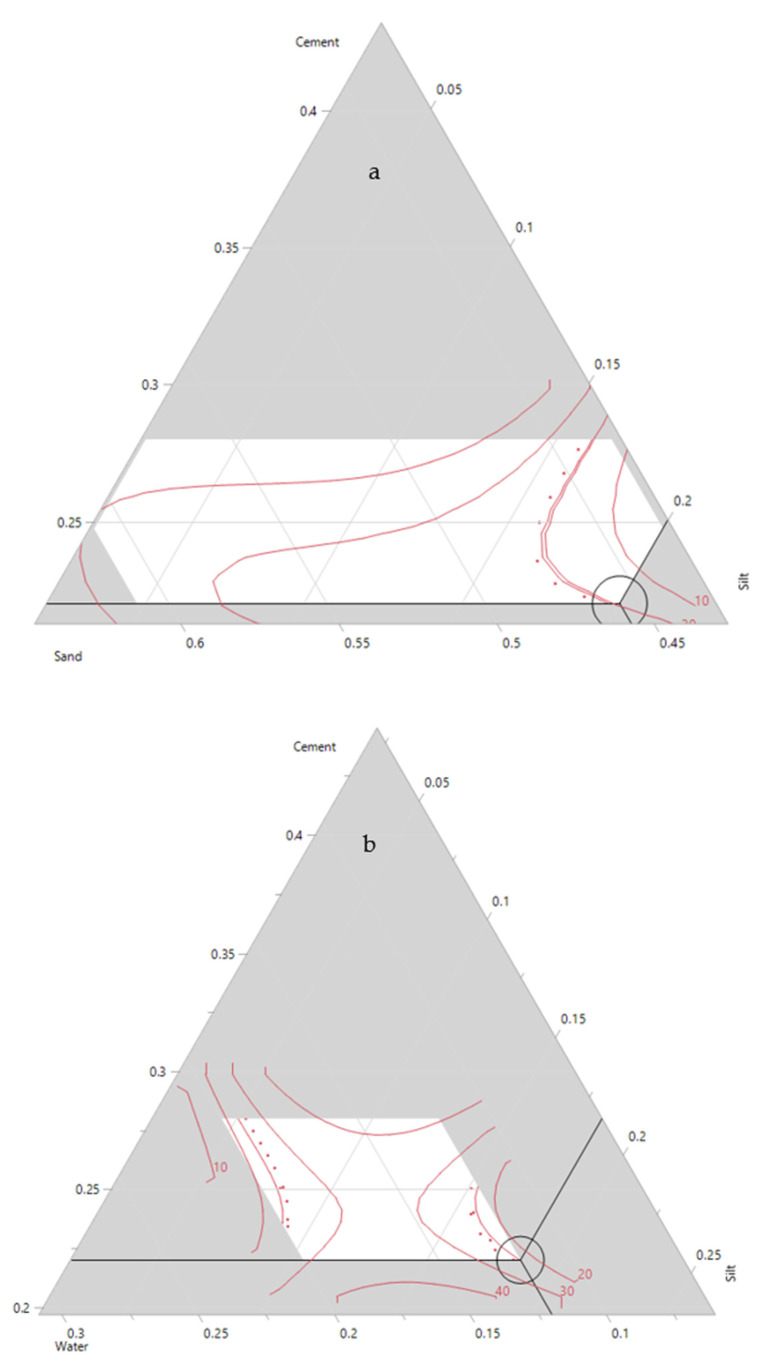
Ternary contour plots for 28 days compressive strength comparing component ratios: (**a**) cement–silt–sand; (**b**) cement–silt–water.

The relationship between silt, sand, cement, water and additives with compressive strength are shown in Figure 8. The highest value for compressive strength is only achieved when the silt amount is minimum (2%). However, the addition of 20% silt and 22% cement could produce mortars with approximately 20 MPa compressive strength (Figure 8a). The silt and sand showed a negative correlation, where a decrease in silt content increased the amount of sand in the mixture. In addition, an increase in sand content led to an increase in compressive strength (Figure 8b). The effect of sand and cement on the compressive strength is also shown in the ternary plots (Figure 9a), where an increase in sand and cement content increases the final compressive strength. The highest UCS values were observed when the sand ratio was between 56 to 58% of the total weight of the mortar. The interaction between silt and the additive was not as significant as the interactions between silt and other components. For instance, based on Figure 8c, in a few regions, the decrease or increase of additives did not dramatically affect the overall UCS. An increase in water content (12 to 20%) led to a decrease in the compressive strength of the samples (Figure 8d). This was also confirmed in Figure 9b and is a well-known phenomenon since an increase in water content reduces the compaction rate of the cement/concrete mixtures leading to lower compressive strength.

**Figure 10:**
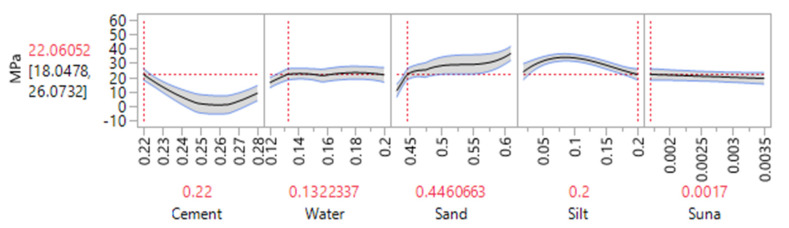
Optimization of 28 days compressive strength based on maximized silt content.

**Figure 11:**
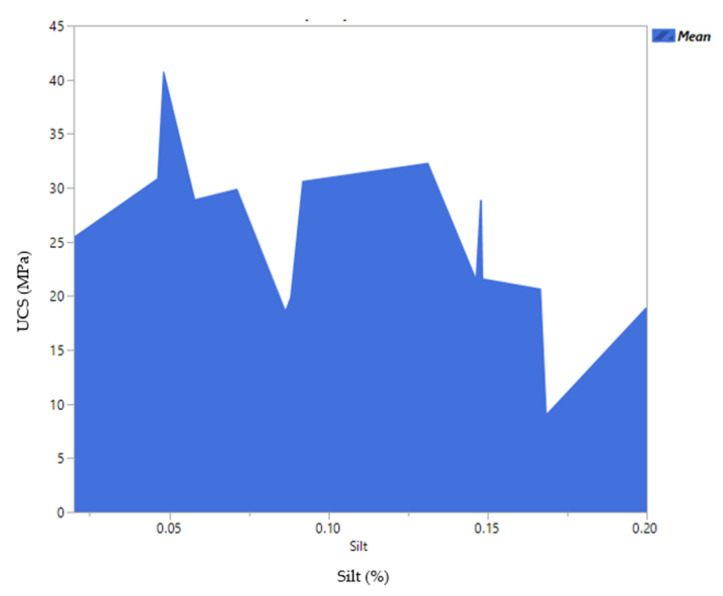
Mean values for 28 days UCS (MPa) versus silt content (%).

One of the aims of conducting a DOE is to benefit from its optimization capabilities. During an optimization process, one could aim for maximizing or minimizing a certain response. Moreover, the optimization could be achieved by defining a certain range or limits. During the optimization process, it is also possible to select the best mixture based on a desirable dependent variable. For instance, the profiler option in JMP software was used and the amount of silt was set to 20%. As illustrated in Figure 10, a compressive strength of 22 MPa could be achieved when the mixture contains 22% cement, 13% water, 44 sand, 0.17% additive and 20% of silt. Decreasing the amount of silt to 16% could increase the compressive strength to 26 MPa. However, since the focus of the study was to maximize the use of waste silt, the model was optimized based on the highest amount of silt in the mixture. The real mean values obtained for a different amount of silt is depicted in Figure 11, where the maximum strength was obtained when only 5% of waste silt was introduced into the mixture.

### 4.2. Flexural Strength

Based on the Anova analysis, a change in the components including cement, water, sand, silt and additive significantly affected the flexural strength of the cement mortars (*p* < 0.0001) (Table 7). The t-ratio reported in Table 8 indicates the influence of each component on the resulting flexural strength values. Compared to other main components, the sand has the highest effect on the mechanical properties, followed by cement. Water had a high and negative correlation with flexural strength. Silt and additive do not have a significant effect on the final response. However, the interaction between water-silt is high and comparable to the effect of sand. Thus, the maximized value for the response will be obtained by maximizing the sand component in the mixture. As mentioned before, despite their insignificance, the silt and additive could not be deleted from the model because of the nature of the mixture design.

**Figure 12:**
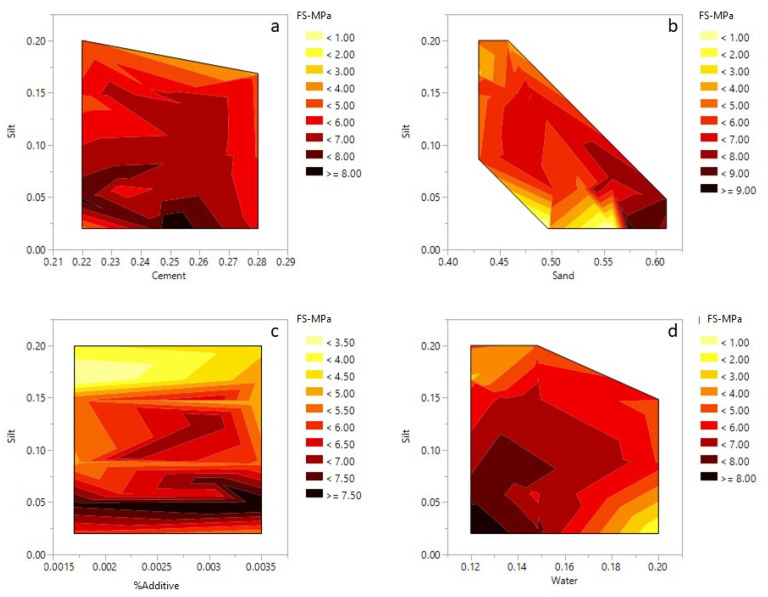
Contour plots for 28 days flexural strength: (**a**) silt–cement; (**b**) silt–sand; (**c**) silt–admixture; (**d**) silt–water.

**Figure 13:**
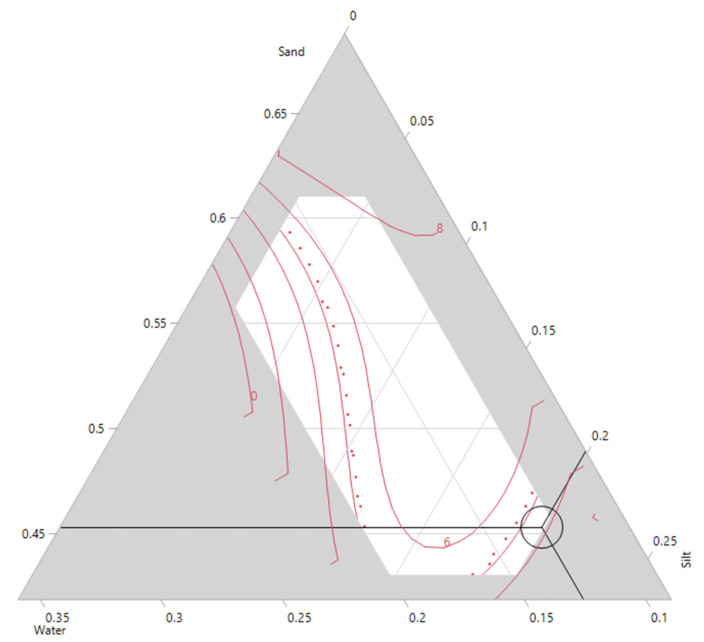
Ternary contour plot for 28 days flexural strength.

The relationship between silt, sand, cement, water and additive with the flexural strength are shown in Figure 12. Like the compressive strength, the highest value for flexural strength is only achieved when the silt amount is set to a minimum (2%). However, the addition of 20% silt and 22% cement could produce mortars withstanding approximately 4.0 MPa of flexural strength (Figure 12a). With the silt content fixed at 2%, the flexural strength decreased when the sand content increased from 50 to 55%. This could be due to the higher content of cement in the mixture (Figure 12b). However, when the amount of silt is increased to 10% or higher, an increase of sand content in the mortar mixtures improves the flexural strength. An increase in the additive content improved the flexural strength, which is visible in the contour plot (Figure 12c). Regardless of silt content, the flexural values improve with higher additive values. However, this trend reverses and the flexural strength starts to decrease for additive values higher than 0.3%. Similar trends were observable for the model constructed for the compressive strength. The effects of water, sand and silt on the flexural strength are also observable in the ternary plot (Figure 13). The increase of silt follows a similar trend to water and decreases the flexural strength of the mixture. However, it is only the sand that improves the final strength of the mixture. 

**Figure 14:**
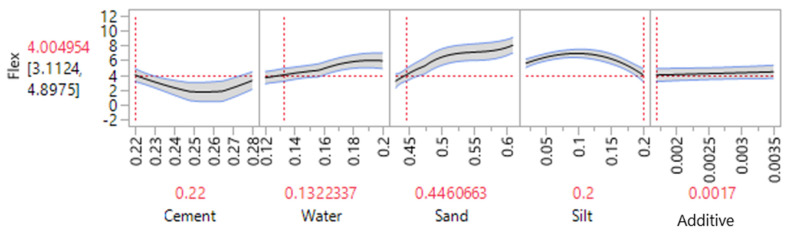
Optimization of 28 days flexural strength (MPa) based on maximized silt content.

**Figure 15:**
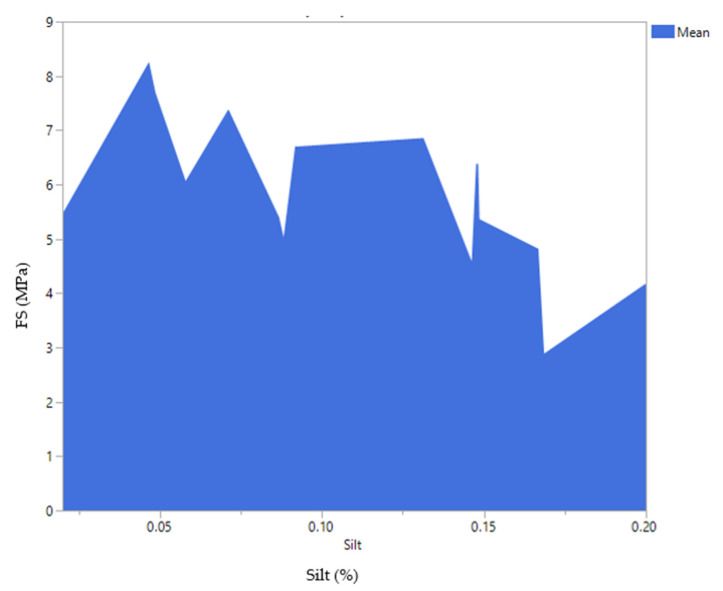
Mean values for 28 days flexural strength (FS) (MPa) versus silt content (%).

The profiler option in JMP software was used and the silt was set to 20 percent. As illustrated in Figure 14, a flexural strength of 4.0 MPa could be achieved when the mixture contains 22% cement, 13% water, 44.83% sand, 0.17% additive and 20% of silt. Decreasing the amount of silt to 16% could increase the strength to 6 MPa. However, as highlighted before, the current work aimed to improve the silt content without compromising the cement mortar performances. Water to cement ratios for both responses was approximately 0.59 which is slightly higher than the normal values (*W*/*C* = 0.5). Due to the nature of silt/clay particles extra water is required to increase the workability of the cement mortar mixtures [29]. The real mean values obtained for different amounts of silt is depicted in Figure 15, where the maximum strength was obtained when only 5% of waste silt was introduced into the mixture. The overall trend indicates and confirms a decrease in the strength with an increase of silt content in the mortar mixtures. 

## 5. Conclusions

The current research focused on producing cement mortars by partially substituting natural sand with the waste silt obtained from the limestone aggregate production in S.A.P.A.B.A. s.r.l. (Italy). Thus, a DOE method was proposed to define the optimum mix design, aiming to include waste silt without affecting the final performance of the cement mortar. Three cement mortar beams were produced and tested for each of the 49 randomized mixtures defined by the DOE method. The obtained results validate the design approach and suggest the possibility of substituting up to 20% of natural sand with waste silt in cement mortar mixtures. The corresponding maximum compressive and flexural strength were reported as 22 MPa and 4.0 MPa, respectively. However, by reducing the silt amount to 16%, the compressive and flexural strength will increase to approximately 30 and 6.5 MPa, respectively. The silt particles are larger than natural sand and shorter than clay minerals. When silt and clay particles are introduced into cement/concrete mixtures, more water is required for producing a homogenized mixture. However, the addition of too much water could decrease the compaction of the mixture and reduce the final strength of the mixture. The addition of a special admixture improved the mixing process of the mixture even though it showed no significant effects on the models. Recycling a high amount of washed silt into cement-bound materials could dramatically help with the recycling process of such material and decrease its adverse impact on the environment.

## Figures and Tables

**Table 1:** Chemical oxides present in waste silt.

Parameter	Value (%)
SiO_2_	43.5
TiO_2_	0.6
Al_2_O_3_	12.5
Fe_2_O_3_	6.1
MnO	0.2
CaO	15.8
Na_2_O	1.0
K_2_O	1.9
P_2_O_5_	0.1
MgO	3.0

**Table 2:** Summary of fit. Effect of components on Unconfined Compressive Strength (UCS).

Indicator	Value
R Square	0.955208
R Square Adj	0.925996
Root Mean Square Error	3.106147
Mean of Response	25.78675

**Table 3:** Summary of fit. Effect of components on flexural strength.

Indicator	Value
R Square	0.911509
R Square Adj	0.879905
Root Mean Square Error	0.795718
Mean of Response	5.679888
Observations (or Sum Wgts)	39

**Table 4:** Design of experiments data.

	Cement (%)	Water (%)	Sand (%)	Silt (%)	Additive (%)	FS (MPa)	UCS (MPa)	Pred. FS (MPa)	Pred. UCS (MPa)
1	22.00	20.00	55.65	2.00	0.35	0.00	0.00	−0.31	0.60
2	22.00	14.65	43.00	20.00	0.35	3.83	18.17	4.16	16.95
3	24.64	13.56	55.79	5.79	0.215	6.08	30.01	6.92	31.50
4	28.00	20.00	43.00	8.83	0.17	4.91	19.86	5.70	22.73
5	24.83	12.00	61.00	2.00	0.17	9.04	34.59	8.06	36.96
6	22.00	14.83	43.00	20.00	0.17	4.51	18.94	3.94	19.23
7	22.00	12.00	45.65	20.00	0.35	4.88	20.95	4.37	17.83
8	22.00	12.00	61.00	4.65	0.35	8.20	30.82	8.68	34.47
9	28.00	20.00	43.00	8.65	0.35	5.39	18.45	4.38	14.57
10	28.00	20.00	49.83	2.00	0.17	3.91	16.05	3.03	11.78
11	23.23	13.56	48.21	14.79	0.215	6.45	27.41	6.43	28.84
12	22.00	14.83	61.00	2.00	0.17	7.19	32.48	6.60	30.93
13	24.55	13.56	46.79	14.79	0.305	5.20	30.78	6.36	26.95
14	23.23	17.56	53.12	5.79	0.305	4.77	25.15	4.19	23.09
15	26.23	17.56	46.79	9.21	0.215	6.68	30.55	6.47	31.59
16	22.00	12.00	45.83	20.00	0.17	3.40	17.48	3.95	21.04
17	23.23	13.56	48.12	14.79	0.305	7.07	30.66	6.65	28.65
18	26.23	13.56	46.79	13.12	0.305	6.84	32.21	7.21	33.33
19	28.00	12.00	43.00	16.83	0.17	2.85	8.89	3.26	8.95
20	22.00	20.00	43.00	14.65	0.35	4.49	21.32	4.59	21.30
21	22.00	12.00	61.00	4.83	0.17	7.70	40.45	8.04	36.64
22	28.00	12.00	43.00	16.65	0.35	4.80	20.59	3.95	21.84
23	23.23	14.98	55.79	5.79	0.215	6.72	32.31	5.93	28.51
24	22.00	20.00	43.00	14.83	0.17	5.35	21.53	5.93	21.70
25	23.23	17.56	53.21	5.79	0.215	5.20	25.96	4.34	23.66
26	26.23	17.56	50.12	5.79	0.305	6.02	29.26	5.47	27.90
27	23.23	14.98	46.79	14.79	0.215	6.02	26.86	6.33	29.60
28	22.00	14.65	61.00	2.00	0.35	7.46	29.35	7.15	30.08
29	24.64	13.56	46.79	14.79	0.215	6.25	28.46	6.13	25.89
30	23.23	14.89	46.79	14.79	0.305	6.95	27.17	6.47	29.18
31	28.00	12.00	57.65	2.00	0.35	10.31	55.91	9.81	54.70
32	23.23	13.56	55.79	7.12	0.305	7.34	29.80	6.99	30.37
33	28.00	12.00	57.83	2.00	0.17	8.87	43.96	9.17	42.33
34	26.23	17.56	50.21	5.79	0.215	5.98	24.62	5.62	28.73
35	23.23	14.89	55.79	5.79	0.305	5.63	25.05	6.15	28.45
36	26.23	13.56	54.12	5.79	0.305	7.77	38.37	7.96	39.10
37	28.00	20.00	49.65	2.00	0.35	0.00	0.00	1.79	3.86
38	24.65	12.00	61.00	2.00	0.35	7.48	41.25	8.69	41.14
39	22.00	20.00	55.83	2.00	0.17	0.00	0.00	0.96	0.70

FS: flexural strength; UCS: unconfined compressive strength; pred flex: predicted values for flexural strength; pred UCS: predicted values for UCS.

**Table 5:** Analysis of variance for the UCS model.

Source	DF	Sum of Squares	Mean Square	F Ratio
Model	15	4732.2927	315.486	32.6991
Error	23	221.9074	9.648	Prob > F
U. Total	38	4954.2001	-	<0.0001 *

**Table 6:** Effect summary/parameter estimates for compressive strength values.

Term	Estimate	Std Error	t Ratio	Prob > |t|
(Cement-0.22)/0.2083	85.702806	17.88592	4.79	<0.0001 *
(Water-0.12)/0.2083	−62.6514	38.50179	−1.63	0.1173
(Sand-0.43)/0.2083	48.611973	4.193305	11.59	<0.0001 *
(Silt-0.02)/0.2083	9.0094789	3.948727	2.28	0.0321 *
(Additive-0.0017)/0.2083	−282.3226	278.2153	−1.01	0.3208
Cement * Water	−11.66381	88.82965	−0.13	0.8967
Water * Sand	−21.87772	67.99387	−0.32	0.7505
Water * Silt	169.97529	66.1488	2.57	0.0171 *
Sand * Silt	0.2183764	21.45658	0.01	0.9920
Cement * Additive	5901.5652	1620.775	3.64	0.0014 *
Water*Additive	808.67954	1254.483	0.64	0.5255
Cement * Sand * (Cement-Sand)	195.20254	62.03723	3.15	0.0045 *
Cement * Silt * (Cement-Silt)	254.78546	63.84024	3.99	0.0006 *
Water * Sand * Silt	409.27446	187.3776	2.18	0.0394 *
Sand * Silt * (Sand-Silt)	−77.41989	18.82222	−4.11	0.0004 *
Cement * Water * Additive	−23,724.89	6424.449	−3.69	0.0012 *

Statistically significant parameters are indicated by *.

**Table 7:** Analysis of variance for flexural strength model.

Source	DF	Sum of Squares	Mean Square	F Ratio
Model	10	182.61511	18.2615	28.8415
Error	28	17.72870	0.6332	Prob > F
U. Total	38	200.34381	-	<0.0001 *

**Table 8:** Effect summary/parameter estimates for flexural strength values.

Term	Estimate	Std Error	t Ratio	Prob > |t|
(Cement-0.22)/0.2083	17.270749	1.904416	9.07	<0.0001 *
(Water-0.12)/0.2083	−13.02035	1.903995	−6.84	<0.0001 *
(Sand-0.43)/0.2083	9.6833449	0.748193	12.94	<0.0001 *
(Silt-0.02)/0.2083	0.5155091	0.79756	0.65	0.5233
(Additive-0.0017)/0.2083	73.118157	45.426	1.61	0.1187
Water * Silt	44.859904	4.34842	10.32	<0.0001 *
Sand * Silt	7.619343	2.810101	2.71	0.0113 *
Cement * Sand * (Cement-Sand)	31.001789	10.3192	3.00	0.0056 *
Cement * Silt * (Cement-Silt)	23.954223	10.01144	2.39	0.0237 *
Sand * Silt * (Sand-Silt)	−15.11265	4.698824	−3.22	0.0033 *
Water * Additive * (Water-Additive)	−1462.162	549.0786	−2.66	0.0127 *

Statistically significant parameters are indicated by *.

## Data Availability

All data has been provided within the article.

## Supplementary Materials

The following are available online at https://www.mdpi.com/1996-1944/14/3/528/s1, Equation 1S: Predicted model for FS, Equation 2S: Predicted model for UCS.

## References

[1] European Commission (1999). Council Directive 1999/31/EC of 26 April 1999 on the landfill of waste. Off. J. Eur. Communities.

[2] European Union, UEPG-European Aggregates Association (2020). A Sustainable Industry for a Sustainable Europe. Annual Review. https://uepg.eu/mediatheque/media/UEPG-AR20192020_V13_(03082020)_spreads.pdf.

[3] Eurostat (2020). Generation of Waste by Waste Category, Hazardousness and NACE Rev. 2 Activity (env_wasgen). https://appsso.eurostat.ec.europa.eu/nui/submitViewTableAction.do.

[4] Solouki A., Viscomi G., Lamperti R., Tataranni P. (2020). Quarry Waste as Precursors in Geopolymers for Civil Engineering Applications: A Decade in Review. Materials.

[5] Saghafi B., Al Nageim H., Phil P.V., Ghazireh N. (2012). Use of waste limestone dust and steel slag in UK highways type 1 unbound mixtures. Proc. Inst. Civ. Eng. Constr. Mater..

[6] Do H.S., Mun P.H., Keun R.S. (2008). A study on engineering characteristics of asphalt concrete using filler with recycled waste lime. Waste Manag..

[7] Puppala A.J., Saride S., Williammee R. (2011). Sustainable Reuse of Limestone Quarry Fines and RAP in Pavement Base/Subbase Layers. J. Mater. Civ. Eng..

[8] Chang F.C., Lee M.Y., Lo S.L., Lin J.D. (2010). Artificial aggregate made from waste stone sludge and waste silt. J. Environ. Manag..

[9] Cavaleri L., Borg R.P., la Mantia F.P., Liguori V. (2018). Quarry limestone dust as fine aggregate for concrete. IOP Conf. Ser. Mater. Sci. Eng..

[10] Medina G., del Bosque I.F.S., Frías M., de Rojas M.I.S., Medina C. (2018). Durability of new recycled granite quarry dust-bearing cements. Constr. Build. Mater..

[11] Felekoglu B. (2007). Utilisation of high volumes of limestone quarry wastes in concrete industry (self-compacting concrete case). Resour. Conserv. Recycl..

[12] Uysal M., Yilmaz K. (2011). Effect of mineral admixtures on properties of self-compacting concrete. Cem. Concr. Compos..

[13] Benarchid Y., Taha Y., Argane R., Benzaazoua M. (2018). Application of Quebec recycling guidelines to assess the use feasibility of waste rocks as construction aggregates. Resour. Polic..

[14] Kitouni S., Houari H. (2014). Lightweight concrete with Algerian limestone dust: Part I: Study on 30% replacement to normal aggregate at early age. Cerâmica.

[15] Shi C., Wu Z., Lv K., Wu L. (2015). A review on mixture design methods for self-compacting concrete. Constr. Build. Mater..

[16] Yildizel S.A., Tayeh B.A., Calis G. (2020). Experimental and modelling study of mixture design optimisation of glass fibre-reinforced concrete with combined utilisation of Taguchi and Extreme Vertices Design Techniques. J. Mater. Res. Technol..

[17] Abouhussien A.A., Hassan A.A.A. (2015). Optimizing the durability and service life of self-consolidating concrete containing metakaolin using statistical analysis. Constr. Build. Mater..

[18] Chen S.-H., Chang C.-S., Wang H.-Y., Huang W.-L. (2011). Mixture design of high performance recycled liquid crystal glasses concrete (HPGC). Constr. Build. Mater..

[19] Rezaifar O., Hasanzadeh M., Gholhaki M. (2016). Concrete made with hybrid blends of crumb rubber and metakaolin: Optimization using Response Surface Method. Constr. Build. Mater..

[20] Ferdosian I., Camões A. (2017). Eco-efficient ultra-high performance concrete development by means of response surface methodology. Cem. Concr. Compos..

[21] Cotto-Ramos A., Dávila S., Torres-García W., Cáceres-Fernández A. (2020). Experimental design of concrete mixtures using recycled plastic, fly ash, and silica nanoparticles. Constr. Build. Mater..

[22] Bouziani T. (2013). Assessment of fresh properties and compressive strength of self-compacting concrete made with different sand types by mixture design modelling approach. Constr. Build. Mater..

[23] de Matos P.R., Sakata R.D., Gleize P.J.P., de Brito J., Repette W.L. (2020). Eco-friendly ultra-high performance cement pastes produced with quarry wastes as alternative fillers. J. Clean. Prod..

[24] Schankoski R.A., de Matos P.R., Pilar R., Prudêncio L.R., Ferron R.D. (2020). Rheological properties and surface finish quality of eco-friendly self-compacting concretes containing quarry waste powders. J. Clean. Prod..

[25] Polydorou T., Constantinides G., Neocleous K., Kyriakides N., Koutsokeras L., Chrysostomou C., Hadjimitsis D. (2020). Effects of pre-treatment using waste quarry dust on the adherence of recycled tyre rubber particles to cementitious paste in rubberised concrete. Constr. Build. Mater..

[26] Tataranni P. (2019). Recycled Waste Powders for Alkali-Activated Paving Blocks for Urban Pavements: A Full Laboratory Characterization. Infrastructures.

[27] Box G.E.P., Wilson K.B. (1951). On the Experimental Attainment of Optimum Conditions. J. R. Stat. Soc. Ser. B.

[28] Smiti A. (2020). A critical overview of outlier detection methods. Comput. Sci. Rev..

[29] Nehdi M.L. (2014). Clay in cement-based materials: Critical overview of state-of-the-art. Constr. Build. Mater..

