# Supplementary material for: Preliminary Evaluation of Cement Mortars Containing Waste Silt Optimized with the Design of Experiments Method
Source: Materials (Basel). 2021 Jan 22;14(3):528. doi: 10.3390/ma14030528 (PMC7865585; doi:10.3390/ma14030528)
Supplement: Supplementary file 1 [file materials-14-00528-s001.pdf]

materials

Supplementary Materials

Preliminary Evaluation of Cement Mortars Containing Waste Silt Optimized with the Design of Experiments Method

Abbas Solouki [1,2,*], Giovanni Viscomi [2], Piergiorgio Tataranni [1,*] and Cesare Sangiorgi [1]

[1] Department of Civil, Chemical, Environmental and Materials Engineering, University of Bologna, 40136 Bologna, Italy; Cesare.sangiorgi4@unibo.it
[2] S.A.P.A.B.A. srl, 40037 Pontecchio Marconi, Italy; g.viscomi@sapaba.it
* Correspondence: abbas.solouki2@unibo.it (A.S.); piergiorg.tataranni2@unibo.it (P.T.)

Equation 1S. Predicted model for FS

$$Y_{FS} = 17.27\left(\frac{A-0.22}{0.2083}\right) - 13.02\left(\frac{B-0.12}{0.2083}\right) + 9.68\left(\frac{C-0.43}{0.2083}\right) + 0.51\left(\frac{D-0.02}{0.2083}\right) + 73.11\left(\frac{E-0.0017}{0.2083}\right) + \left(\frac{\left(\frac{B-0.12}{0.2083}\right)\times(D-0.02)}{0.2083}\right)\times 44.85 + \left(\frac{\left(\frac{C-0.43}{0.2083}\right)\times(D-0.02)}{0.2083}\right)\times 7.61 +$$
$$\left(\frac{\left(\frac{A-.22}{0.2083}\right)\times(C-0.43)}{0.2083}\right)\times 31.00\times\left(\frac{A-0.22}{0.2083} - \frac{C-0.43}{0.2083}\right) + \left(\frac{\left(\frac{A-0.22}{0.2083}\right)\times(D-0.02)}{0.2083}\right)\times 23.95\times\left(\frac{A-0.22}{0.2083} - \frac{D-0.02}{0.2083}\right) + \left(\frac{\left(\frac{C-0.43}{0.2083}\right)\times(D-0.02)}{0.2083}\right)\times(-15.11)\times\left(\frac{C-0.43}{0.2083} - \frac{D-0.02}{0.2083}\right) +$$
$$\left(\frac{\left(\frac{B-0.12}{0.2083}\right)\times(E-0.0017)}{0.2083}\right)\times(-1462.16)\left(\frac{B-0.12}{0.2083} - \frac{E-0.0017}{0.2083}\right)$$

, where A, B, C, D and E represent cement, water, sand, silt and additive, respectively.

Equation 2S. Predicted model for UCS

$$Y_{UCS} = 85.70\times\left(\frac{A-0.22}{0.2083}\right) - 62.65\left(\frac{B-0.12}{0.2083}\right) + 48.61\left(\frac{C-0.43}{0.2083}\right) + 9.00\left(\frac{D-0.02}{0.2083}\right) - 282.32\left(\frac{E-0.0017}{0.2083}\right) + \left(\frac{\left(\frac{A-0.22}{0.2083}\right)(B-0.12)}{0.2083}\right)(-11.66)$$
$$+\left(\frac{\left(\frac{B-0.12}{0.2083}\right)(C-0.43)}{0.2083}\right)(-21.87) + \left(\frac{\left(\frac{B-0.12}{0.2083}\right)(D-0.02)}{0.2083}\right)169.97 + \left(\frac{\left(\frac{C-0.43}{0.2083}\right)(D-0.02)}{0.2083}\right)0.21$$
$$+\left(\frac{\left(\frac{A-0.22}{0.2083}\right)(E-0.0017)}{0.2083}\right)5901.56 + \left(\frac{\left(\frac{B-0.12}{0.2083}\right)(E-0.0017)}{0.2083}\right)808.67$$
$$+195.20\left(\frac{\left(\frac{A-0.22}{0.2083}\right)(C-0.43)}{0.2083}\right)\left(\frac{A-0.22}{0.2083} - \frac{C-0.43}{0.2083}\right) + 254.78\left(\frac{\left(\frac{A-0.22}{0.2083}\right)(D-0.02)}{0.2083}\right)\left(\frac{A-0.22}{0.2083} - \frac{D-0.02}{0.2083}\right)$$
$$+\left(\frac{\left(\frac{\left(\frac{B-0.12}{0.2083}\right)(C-0.43)}{0.2083}\right)(D-0.02)}{0.2083}\right)409.27 - 77.41\left(\frac{\left(\frac{C-0.43}{0.2083}\right)(D-0.02)}{0.2083}\right)\left(\frac{C-0.43}{0.2083} - \frac{D-0.02}{0.2083}\right)$$
$$-2372489\left(\frac{\left(\frac{\left(\frac{A-0.22}{0.2083}\right)(B-0.12)}{0.2083}\right)(E-0.0017)}{0.2083}\right)$$

, where A, B, C, D and E represent cement, water, sand, silt and additive, respectively.
